# The Effects of Buckwheat Leaf and Flower Extracts on Antioxidant Status in Mouse Organs

**DOI:** 10.1155/2018/6712407

**Published:** 2018-06-14

**Authors:** Ilona Sadauskiene, Arunas Liekis, Rasa Bernotiene, Jurgita Sulinskiene, Arturas Kasauskas, Gediminas Zekonis

**Affiliations:** ^1^Neuroscience Institute, Lithuanian University of Health Sciences, Kaunas, Lithuania; ^2^Department of Biochemistry, Medical Academy, Lithuanian University of Health Sciences, Kaunas, Lithuania; ^3^Department of Prosthodontics, Medical Academy, Lithuanian University of Health Sciences, Kaunas, Lithuania

## Abstract

This study was undertaken to investigate the effects of the extracts of buckwheat leaf and flower on the antioxidant status of the brain and liver tissue. The administration of buckwheat extracts (both concentrations were 10%) to mice (at the dose 10 mL/kg of body weight) for 21 days significantly decreased superoxide dismutase (SOD) activity and reduced the amount of glutathione (GSH) and malondialdehyde (MDA) in the mouse brain, while catalase (CAT) activity significantly increased. In the mouse liver, the amount of GSH and activity of SOD increased, while the CAT activity after administering buckwheat leaf and flower extracts was lower in experimental mice than in the control group. However, the administration of 10% ethanol (for 21 days) to control animals also had a significant effect on the antioxidant system in brain and liver cells. Experimental animals demonstrated rather marked changes in the activities of the antioxidant enzymes SOD and CAT in their liver and brain cells, and changes in the levels of GSH and MDA were observed when compared with the control group.

## 1. Introduction

Buckwheat (*Fagopyrum esculentum* Moench) is an herbaceous plant that belongs to the Polygonaceae family. This plant is known as a dietary source of protein with favorable amino acid composition, fibers, vitamins (B_1_ and B_2_), starch, essential minerals, and trace elements [1–3]. Buckwheat grains and hulls contain biologically active components, such as flavonoids and flavones, tannins, phytosterols, and fagopyrins. Flavonoids act as antioxidants inhibiting lipid peroxidation and attenuating damage inflicted by reactive oxygen species [4, 5]. A number of studies have shown that buckwheat possesses strong antioxidant activity, mainly due to high rutin content [6, 7]. Flavonoids in buckwheat decrease blood cholesterol levels helping to prevent high blood pressure. Rutin, composed of flavonol quercetin and disaccharide rutinose, has an anti-inflammatory and hypotensive effect. Furthermore, rutin/quercetin inhibits oxidation of lipoproteins, which suggests that rutin reduces the risk for arteriosclerosis [8]. It should be noted that rutin content of the flower part is higher than that of other parts of buckwheat—leaf, seeds, or roots. About 10% of rutin (per dry weight) is found in buckwheat flower and leaf [5].

It is noteworthy that in practice, mostly ethanol extracts of buckwheat leaf and flower are used as medicinal preparations. However, ethanol itself affects body cells, and this effect in most cases is defined as harmful. As it is known, ethanol is a nonelectrolyte that is comparatively quickly absorbed from the gastrointestinal tract, diffuses into circulation, and is distributed uniformly throughout the body. In the body, ethanol is metabolized in enzyme-catalyzed oxidative processes. Initially forming acetaldehyde, it is further oxidized to acetate, which is then converted to carbon dioxide via the citric acid cycle. It is important that ethanol and its metabolites can also cause autooxidation of the cells of the body organs (e.g., hepatic, brain, and kidney cells) either by acting as a prooxidant or by reducing the antioxidant levels and resulting in marked hepatotoxicity or damage to other organs. Lipid peroxidation and the associated membrane damage are key features in alcohol-induced cell injury. The protective action of antioxidants is due to the inhibition of free radical-induced chain reaction and the resultant prevention of oxidative deterioration of structural lipids in membranous organelles. The ability of organ cells to resist oxidative stress, which causes oxidative damage, is largely dependent on the efficiency of the overall antioxidant defense system [9]. This antioxidant defense network consists of endogenous and exogenous elements. The endogenous elements comprise enzymatic antioxidants such as SOD, CAT, and GPx (glutathione peroxidase). This group also includes nonenzymatic antioxidants: GSH and vitamins C and E as well as small molecules. The exogenous antioxidants comprise micronutrients and other exogenously administered antioxidants. An antioxidant is defined as any substance that delays, prevents, or removes oxidative cell damage. As indicated before, buckwheat has an abundance of such substances, as does its ethanol extract used in medicine. However, it is still not entirely clear what effect the substances found in buckwheat flower and leaf have on the functioning of the cell's antioxidant system and how this system is affected by ethanol, which forms the basis of the extracts. The literature survey showed that its mechanisms of action have not been studied enough.

The present study was planned to evaluate the effects of buckwheat flower and leaf extracts as well as ethanol used in the production of the extracts on the antioxidant system in liver and brain cells.

## 2. Materials and Methods

### 2.1. The Object of Research

The experiments were done on 4–6-week-old outbred white laboratory mice weighing 20–25 g. All experiments were performed according to the Republic of Lithuania Law on Welfare and Protection of Animals (License of the State Food and Veterinary Service for working with laboratory animals number G2-19). Enzyme activities as well as MDA and GSH concentrations in mouse organs were determined after 21-day intragastric administration of buckwheat extracts. Each of two experimental groups consisted of 10 mice. The studied substances were administered directly into the stomach of the mice via an intragastric feeding tube. The concentration of buckwheat leaf and flower extracts was 10%; the volume of the extracts administered was adjusted for the weight of mice based on the ratio of 10 mL/kg body weight. Control animals (10 mice) received the same volume of saline (control 1 group) using the same method of administration. Buckwheat extracts we used were alcohol-based, so an additional control group (control 2 group) received the same volume of 10% ethanol (10 mice). After the exposure time, the animals were terminated according to the rules defined by the European Convention for the Protection of Vertebrate Animals Used for Experimental and Other Purposes. Following that, the brain and liver were removed, put on Petri dishes, and immediately cooled in an ice bath.

### 2.2. Preparation of Plant Samples

Buckwheat *Fagopyrum esculentum* Moench (“VB Noja,” originating from Lithuania) leaf and flower extracts used in our experiments were obtained from the Department of Analytical and Toxicological Chemistry of the Lithuanian University of Health Sciences. Dried buckwheat samples of aerial parts were ground in a laboratory mill GM 300 (Retsch, Germany). Subsequently, 1 g of the obtained powder was extracted under optimized extraction conditions with 10 mL of 96% ethanol in an ultrasonic bath for 15 min at 45°C. The extract was then centrifuged for 10 min at 6000 rpm, which was followed by supernatant collection. Samples were filtered through membrane filters (pore size 0.22 *μ*m).

Based on the data of our buckwheat leaf and flower extract suppliers [10] and literature [6, 11–14], biologically active compounds were identified in the buckwheat leaf and flower extracts. The list of the compounds and their concentrations is provided in Table 1.

### 2.3. Measurement of GSH Amount in Mouse Organs

GSH content was evaluated using the method described by Bernotiene et al. [15]. The removed mouse brain/liver was weighed and homogenized in 6 volumes (as compared with tissue weight) of 5% trichloroacetic acid solution. The homogenate was centrifuged at 10,000 ×g for 7 min to obtain the GSH-containing supernatant. GSH was measured by reaction with *DTNB* (also called Ellman's reagent or 5,5′-dithiobis(2-nitrobenzoic acid)) to form a compound that absorbs light of 412 nm wavelength. Each sample cuvette contained 2 mL of 0.6 mM DTNB in 0.2 M sodium phosphate, pH 8.0; 0.2 mL of supernatant fraction; and 0.8 mL of 0.2 M phosphate buffer to the final volume of 3 mL. GSH content was expressed as *μ*mol/g of the wet weight of the tissue.

### 2.4. Measurement of MDA Amount in Mouse Organs

The final product of lipid peroxidation, MDA, forms a complex with TBA (thiobarbituric acid), and the content of it can be determined spectrophotometrically; the results are expressed as nmol/g of the wet weight of the tissue [15]. The brain/liver was removed and homogenized with 9 volumes (as compared with tissue weight) of cold 1.15% KCl to make 10% homogenate. Subsequently, 3 mL of 1% H_3_PO_4_ and 1 mL of 0.6% TBA aqueous solution were added to 0.5 mL of this homogenate. The mixture was heated for 45 min in a boiling water bath. After cooling, 4 mL of n-butanol was added and the resulting mixture was stirred vigorously. The butanol phase was separated by centrifugation, and supernatant absorbance was determined at 535 and 520 nm wavelengths.

### 2.5. CAT Activity Assay

CAT activity in brain and liver homogenates was determined according to the method described by Sadauskiene et al. [16]. The method is based on the decomposition of hydrogen peroxide (H_2_O_2_) by CAT. The reaction mixture was composed of 50 mM Tris-HCl buffer of pH 7.4 with 18 mM H_2_O_2_ (buffer-substrate mixture) and 100 *μ*L of the organ homogenate. The reaction mixture was incubated at 37°C for 180 s. The enzymatic reaction was stopped with 2.0 mL of 4.5% ammonium molybdate, and the absorbance of the yellow complex of molybdate and hydrogen peroxide was measured at 410 nm wavelength against blank (buffer-substrate mixture incubated for 180 s with subsequently added ammonium molybdate and 100 *μ*L of the homogenate). The results were expressed in U/mg protein. One unit of catalase (U) decomposes 1 *μ*mol of hydrogen peroxide per 1 min under these conditions.

### 2.6. SOD Activity Assay

The activity of superoxide dismutase in the brain homogenate was evaluated according to the method described by Rachmanova et al. [17]. The spectrophotometric evaluation of SOD activity is based on the inhibition of nitroblue tetrazolium (4-nitroblue tetrazolium chloride, NBT) reduction rate in the nonenzymatic phenazine methosulfate-nicotinamide adenine dinucleotide system. First, we prepared a reaction mixture consisting of 0.1 M of phosphate buffer (pH 7.8), 0.41 mM of NBT, 0.33 mM of EDTA (ethylenediaminetetraacetic acid), 0.01 mM of PMS (phenazine methosulfate), and 0.8 mM of NADH. For the initiation of the reaction, a selected amount of the brain homogenate was added to this mixture. A spectrophotometer was used to measure optical density at 540 nm—*E*_0_ (initial extinction). After 5 min, the optical density was measured again—EC_B_ (extinction after 5 min). The SOD activity was expressed as U/mg protein, where U was a relative unit of activity defined as the amount of SOD required for the inhibition of NBT reduction by 50% and expressed as a unit of activity in a 1 mg protein sample.

### 2.7. Protein Concentration Assay

Protein concentration in homogenate samples of the brain and liver was measured by using the Warburg-Christian method.

### 2.8. Statistical Analysis

The data were expressed as mean ± SEM (standard error of the mean). Statistical significance was assessed using one-way analysis of variance (ANOVA) and the unpaired Student *t*-test. The value of *p* < 0.05 was considered as statistically significant (SPSS version 19.0, SPSS).

## 3. Results

**Figure 1:**
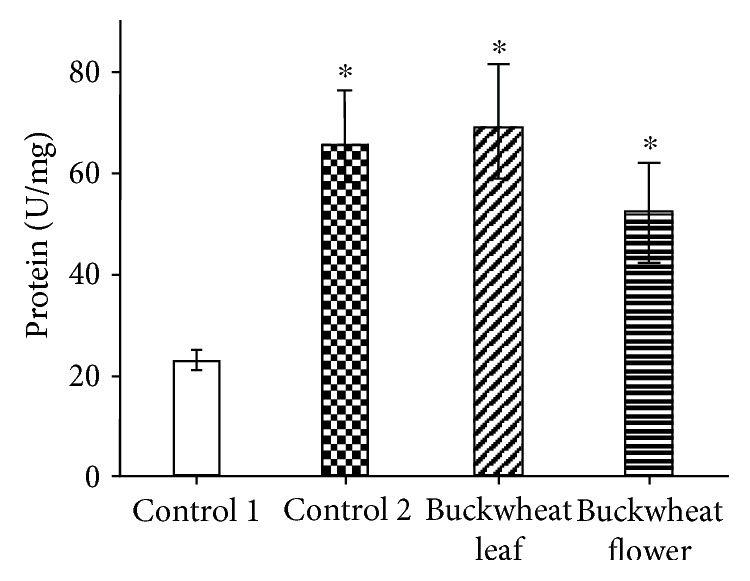
CAT activity in the mouse brain. The presented data are on 8–10 experiments. The mice of the control 1 group received an injection of saline, and the control 2 group mice received ethanol. ^∗^*p* ≤ 0.05, compared with the control 1 group.

**Figure 2:**
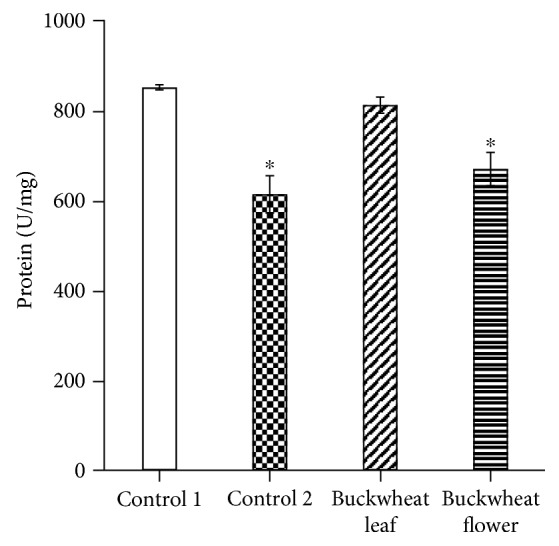
CAT activity in the mouse liver. The presented data are on 8–10 experiments. The mice of the control 1 group received an injection of saline, and the control 2 group mice received ethanol. ^∗^*p* ≤ 0.05, compared with the control 1 group.

**Figure 3:**
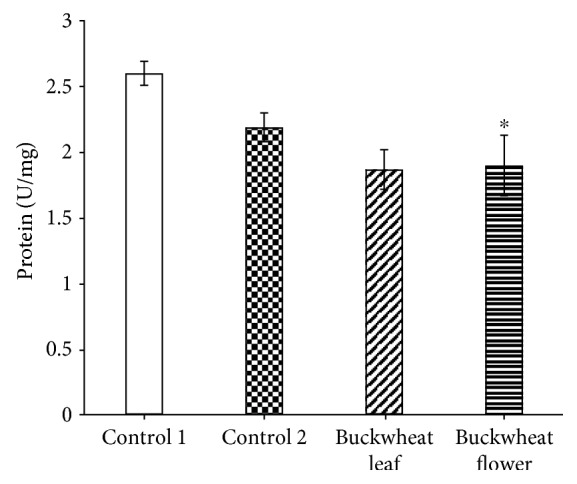
SOD activity in the mouse brain. The presented data are on 8–10 experiments. The mice of the control 1 group received an injection of saline, and the control 2 group mice received ethanol. ^∗^*p* ≤ 0.05, compared with the control 1group.

**Figure 4:**
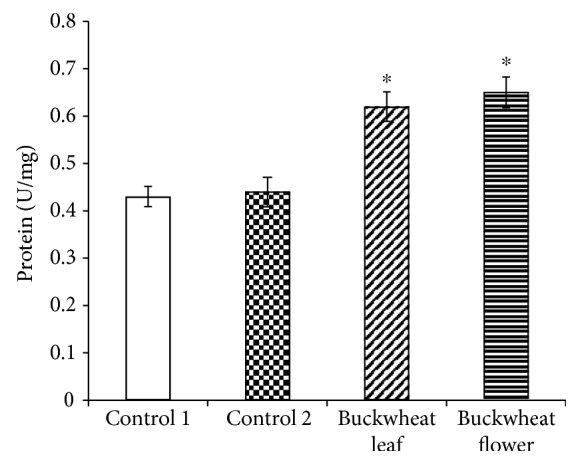
SOD activity in the mouse liver. The presented data are on 8–10 experiments. The mice of the control 1 group received an injection of saline, and the control 2 group mice received ethanol. ^∗^*p* ≤ 0.05, compared with the control 1 group.

Data on the activity of CAT and SOD in the tissues of control and experimental animals are given in Figures 1–4. CAT activity in the brain of mice on ethanol supplementation (control 2) was significantly greater than that of control mice (control 1). In animals treated with buckwheat leaf and flower extracts, CAT activity in the brain was also higher than that in the control 1 group (Figure 1). Meanwhile, CAT activity in the liver of mice that received ethanol (control 2) and buckwheat flower extract was significantly lower than that of control 1 animals (Figure 2). Only buckwheat leaf extract caused no alterations in CAT activity in the liver in comparison with the control 1 group.

The activity of SOD in the brain of mice that received ethanol (control 2) and buckwheat leaf and flower extracts was significantly lower than that in the control 1 group (Figure 3). Meanwhile, this activity in the liver of mice treated with the studied extracts was significantly increased (Figure 4). However, in this case, SOD activity in the control 2 group was the same as that in the control 1 group.

In further experiments, we evaluated the effects of buckwheat leaf and flower extracts on the reduction in the concentration of GSH and MDA in the brain and liver of experimental mice. The concentrations of tissue GSH and MDA in control and experimental animals are given in Table 2.

The obtained results showed that the concentration of GSH was significantly lower in the brain of mice that received buckwheat extracts. GSH concentration in the brain was also lower in mice that were administered only ethanol solution (control 2). However, GSH concentration in the liver of experimental mice was higher compared with that of control 1 mice. MDA level in the brain of the experimental animals was lower than that of the control 1group. However, the liver MDA level did not alter in mice treated with buckwheat leaf and flower extracts as compared with controls (control 1). Meanwhile, in the liver of mice that received only ethanol (control 2), the level of MDA was significantly higher than that in the control 1 group.

## 4. Discussion

The genus *Fagopyrum* has about 15 species distributed in different parts of the world [18]. However, only two types of buckwheat are used as food and medicinal plants in the world: common buckwheat (*Fagopyrum esculentum)* and Tartary buckwheat (*Fagopyrum tataricum)* [19]. It should be noted that buckwheat is grown almost in all continents and that the general composition of crude protein, fiber, fat, and crude ash in both types of buckwheat is essentially the same [2]. Previous research has demonstrated that buckwheat extracts have pharmaceutical effects including antioxidative, antihypertensive, hypolipidemic, and hypoglycemic [20, 21]. In particular, buckwheat extracts demonstrate very high antioxidant activities and are considered to be strong antioxidants that neutralize a wide range of reactive oxygen species and inhibit lipid peroxidation [19, 22, 23]. The antioxidant activities of buckwheat extracts are thought to be related to flavonoids and are believed to play an important role in the alleviation or prevention of many disorders or illnesses. However, little is currently known about the antidisorder and anti-illness effects of buckwheat extracts. Moreover, there is very little information about the effects of buckwheat extracts on various organs of the body when the products are used for a longer period of time in the absence of any pathology. Considering the fact that buckwheat extracts also contain a certain quantity of alcohol, it is very interesting to learn about their influence on the antioxidant system. Thus, the present study was designed to evaluate the effects of buckwheat extracts and ethanol from the extracts on the antioxidant system in normal brain and liver cells of experimental mice.

### 4.1. The Effect of Buckwheat Flower and Leaf Extracts on SOD and CAT Activity in Mouse Brain and Liver

SOD and CAT are called “the first line of defense” in the antioxidant system. These are the enzymes that are found in nearly all living organisms exposed to oxygen. SOD is an enzyme that alternately catalyzes the dismutation of the superoxide radical (O^−^_2_) into molecular oxygen or hydrogen peroxide (H_2_O_2_), while CAT catalyzes the decomposition of hydrogen peroxide into water and oxygen [24]. Both of these enzymes are highly relevant in protecting the body's cells from oxidative damage caused by reactive oxygen species [25]. On the other side, both of these enzymes of the cellular antioxidant system are among the most important biomarkers of oxidative stress. For this reason, in order to determine the state of the antioxidant system in brain and liver cells following the treatment of mice with buckwheat flower and leaf extracts, we decided to evaluate SOD and CAT activities in mouse brain and liver homogenates.

The obtained results showed marked changes in SOD and CAT activities both in the brain and the liver of mice treated for 21 days with the studied preparations (buckwheat flower (BF) and leaf (BL) extracts) and ethanol solution (control 2) compared with control 1 mice. The results also showed that in their trend, these changes differed both between the analyzed enzymes and between the organs. First of all, SOD activity (U) in the brain of the mice affected by the preparations was significantly lower than that observed in control 1 mice (2.59 ± 0.09 U/mg protein): BL—1.86 ± 0.15 U/mg protein (−27%) and BF—1.89 ± 0.23 U/mg protein (−28%). It is interesting to note that SOD activity in mice that received ethanol solution alone was also statistically reliably lower than that in control 1 mice (2.18 ± 0.11 U/mg protein (−17%)). When comparing changes in SOD activity in the brain only between mice affected by BL and BF and the ethanol solution (control 2), the differences were practically nonexistent. Thus, ethanol (the base of the extracts) had a significant effect on SOD activity in brain cells. Analysis of SOD activity in the liver showed that the activity of this enzyme in liver cells of mice that received BL and BF increased statistically reliably, compared with that of control 1 mice (0.43 ± 0.02 U/mg protein). SOD activity in the liver of the mice that received BL reached 0.62 ± 0.03 U/mg protein (+44%, compared with the norm), and in the liver of the mice that received BF, it reached 0.65 ± 0.03 U/mg protein (+51%). In this case, no influence of the ethanol solution on SOD activity was observed (control 2—0.44 ± 0.02 U/mg protein). Thus, the results of our study showed that 21 days of the administration of the studied preparations had a completely different effect on SOD activity in different organs (in this case, in the brain and liver) of experimental mice.

We also evaluated the activity changes of another enzyme of the antioxidant system, CAT, under the same experimental conditions. The results of the evaluation showed that in all cases, CAT activity in the mouse brain increased significantly compared with control 1 mice (22.88 ± 1.98 U/mg protein). Thus, CAT activity in the brain of mice that were administered BL increased up to 201% (68.97 ± 12.32 U/mg protein) and in mice that received BF up to 126% (51.77 ± 7.90 U/mg protein). A very similar increase in CAT activity (186%, 65.51 ± 10.58 U/mg protein) was also observed in the brain of mice that received ethanol solution alone. Meanwhile, completely different changes in CAT activity were observed in the liver of experimental mice. Here, in individual cases, CAT activity had a tendency to decrease (statistically reliably). Thus, CAT activity in the liver of mice that were administered BF was 669.50 ± 36.95 U/mg protein, that is, lower (−21%) than the norm (851.08 ± 5.74), whereas in mice that received ethanol solution, the decrease in the activity of the enzyme was 28% (613.59 ± 41.33 U/mg protein). In our study, only BL had no effect on CAT activity, which was 811.77 ± 17.95 U/mg protein.

We would like to note the fact that the changes in CAT activity under the influence of buckwheat leaf extract and ethanol solution differed radically in different organs (in this case, in the brain and liver). In addition, of special importance is the fact that in cells of the same organs, enzymes of practically the same line, SOD and CAT, demonstrated completely different trends of changes in activity. For instance, while the SOD activity in the mouse brain clearly decreased under the influence of the experimental preparations (including the ethanol solution in control 2 mice), the CAT activity increased. The same picture was observed in the liver of experimental mice, only in this case, the situation was opposite: while the SOD activity increased, the CAT activity decreased. In the case of the liver, two exceptions should be noted: the administration of ethanol solution had no effect on SOD activity, while CAT activity was nearly unaffected by the administration of buckwheat flower extract.

### 4.2. The Effect of Buckwheat Flower and Leaf Extracts on GSH and MDA Amount in Mice Brain and Liver

The other important biomarkers that define oxidation-reduction (redox) state of the cell are changes in GSH and MDA levels. GSH is a major nonprotein thiol in living organisms, which plays a central role in coordinating antioxidant defense processes in the body. It is involved in the maintenance of normal cell structure and function, probably through its redox and detoxification reactions [26]. In our study, we evaluated changes in the amount of GSH in mouse brain and liver cells after 21 days of the administration of BL and BF. The results showed that after 21 days of the administration of the preparations (BL, BF, and ethanol solution (control 2)), the experimental animals demonstrated marked changes in the amount of GSH (compared with control 1 animals) in the brain as well as in the liver. However, while GSH levels in the brain had a tendency to decrease after the administration of the studied preparations, in the liver, they were significantly elevated. Thus, GSH levels in the brain decreased by 42% (after the administration of BL) and 34% (after the administration of BF), compared with control 1 mice. However, practically an equal reduction in GSH levels, 51% compared with control 1 mice, was observed after the administration of ethanol solution alone (control 2). As mentioned before, completely opposite changes in the GSH amount were observed in the mouse liver: GSH levels in the liver of mice that received BL, BF, or ethanol solution increased by 71%, 37%, and 24%, respectively, compared with control 1 mice.

Along with the evaluation of GSH levels, we evaluated MDA level changes in the brain and liver of experimental mice. MDA is a known as the end product of lipid peroxidation and is considered to be a significant marker of the oxidative process in body cells. Our results showed that in the brain of mice that received BL and BF for 21 days, MDA levels decreased statistically reliably, compared with the mice of the control 1 group. In the brain of mice that were administered BL, MDA levels dropped by 32%, while in the brain of mice that received BF, they decreased by 50%. It is noteworthy that MDA levels also clearly decreased (−52%) in the brain of mice of the control 2 group, that is, those that received ethanol solution alone. Meanwhile, MDA levels in the liver of mice that received BL and BF remained virtually unchanged, compared with control 1 mice. However, MDA levels in the liver of control 2 mice (those that received alcoholic solution alone) statistically reliably increased by 22% after 21 days since the initiation of the experiment.

Thus, in general, the results obtained during our experiment showed that BL and BF preparations affected the redox state of mouse organ (brain and liver) cells. After 21 days of the experiment, we observed a clear tendency indicating that the studied preparations inhibited the activity of the enzyme SOD and decreased the amounts of GSH and MDA in mouse brain cells. This effect of buckwheat preparations has been mentioned in the literature as well [27]. However, the results of our study showed that the administration of ethanol solution (the base of the extracts) to mice had practically the same effect. While the data on the inhibition of SOD activity and decrease in GSH levels under the influence of alcohol can be found in literature [28, 29], the reduction in MDA levels observed during our experiment seems to contradict the literature data. In addition, changes in CAT activity in the mouse brain also contradicted the general trend. The activity of this enzyme significantly increased in all the experimental groups.

Opposite changes were observed in the liver of experimental animals. Here, SOD activity and GSH levels increased, whereas MDA levels remained virtually unchanged (except for the control 2 group, where they increased by 22% compared with the control 1 group). In the liver like in the brain, the enzyme CAT demonstrated opposite trends—its activity decreased (except for the group of animals that received BL): in the BF group, it dropped by 21% and in the control group 2 by 28%.

However, one should take into consideration the results obtained using ethanol solution alone (control group 2). In practically all cases, this solution affected the activity of the studied enzymes (SOD and CAT) and the concentrations of GSH and MDA in both brain and liver cells of experimental mice; in the majority of cases, the effect of this reagent was comparable to that of BL and BF. This indicates that the mechanism of action of botanical extracts on the oxidative system of organ cells may be not only that of biologically active substances in the extracts (in this case, BL and BF) but also of the base of the extract, ethanol. Thus, further studies in this area should take into account the effect of individual components of the extract, especially ethanol, on the vital processes in organ cells.

## 5. Conclusions

Our study revealed that repeated administration of buckwheat flower and leaf extracts had an impact on the enzymatic activities of superoxide dismutase and catalase in the brain and liver of mice. A stimulating impact of ethanol on the activities of both enzymes in the organs of the experimental mice was detected as well.

## Figures and Tables

**Table 1:** Biologically active compounds identified in buckwheat leaf and flower extracts.

	Buckwheat leaf	Buckwheat flower
*Phenolic acids*		
Chlorogenic	0.01–0.03%	0.25%
Neochlorogenic	0.3%	0.025%
P-Coumaric	n/d	+
Ferulic	n/d	+
Gallic	n/d	n/d
P-Hydroxybenzoic	n/d	n/d
Fagopyrin	0.05%	0.08%
Tannic	1.0%	5.9%
*Flavonoids*		
Rutin	0.27–10.5%	7.1–12.0%
Quercetin	0.002%	0.005%
Quercitrin	0.02 0.03%	0.35%
Hyperoside	0.005–0.015%	0.04%

n/d: no data.

**Table 2:** Effects of buckwheat leaf and flower extracts on GSH and MDA concentrations in tissues of control and experimental mice.

	GSH (*μ*mol/g tissue)		MDA (nmol/g tissue)	
Groups	Brain	Liver	Brain	Liver
Control 1	2.4 ± 0.06	4.5 ± 0.35	106 ± 2.35	71 ± 2.98
Control 2	1.18 ± 0.02^∗^	5.6 ± 0.17^∗^	51 ± 2.22^∗^	87 ± 4.06^∗^
Buckwheat leaf	1.4 ± 0.02^∗^	7.7 ± 0.27^∗^	73 ± 4.67^∗^	79 ± 4.84
Buckwheat flower	1.6 ± 0.03^∗^	6.2 ± 0.44^∗^	53 ± 0.86^∗^	72 ± 7.44

Values are mean ± SEM (standard error of the mean) of 8–10 mice from each group. ^∗^*p* ≤ 0.05, compared with the control 1 group.

## Data Availability

The data used to support the findings of this study are available from the corresponding author upon request.
